# Structural changes and cellulose ultrastructure mapped with electron microscopy and SAXS after enzymatic hydrolysis of mildly steam pretreated Norway spruce

**DOI:** 10.1186/s13068-025-02616-7

**Published:** 2025-02-21

**Authors:** Maria E. F. Brollo, Fabio Caputo, Polina Naidjonoka, Lisbeth Olsson, Eva Olsson

**Affiliations:** 1 Department of Physics, Chalmers University of Technology, Gothenburg, Sweden; 2 Division of Industrial Biotechnology, Department of Life Sciences, Chalmers University of Technology, Gothenburg, Sweden; 3 Division of Materials Physics, Department of Physics, Chalmers University of Technology, Kemigården 1, 412 96 Gothenburg, Sweden; 4 Wallenberg Wood Science Center, Chalmers University of Technology, Gothenburg, Sweden; 5 Present address: Yangi AB, Varberg, Sweden

**Keywords:** Cellulose, Saccharification, Enzyme accessibility, Hemicellulose, Steam explosion

## Abstract

**Background:**

The efficient use of softwood in biorefineries requires harsh pretreatment conditions to overcome biomass recalcitrance. While this allows the solubilization of hemicellulose, it also leads to the formation of compounds that act inhibitory against microorganisms during the fermentation step. To improve the efficacy of biomass utilization and identify optimal processing conditions, we evaluated the microstructural alterations occurring during pretreatment and enzymatic hydrolysis in Norway spruce. The biomass was steam pretreated at six different severities defined by two different temperatures (180 °C and 210 °C), with and without the addition of various acids (HAc, H_3_PO_4_, H_2_SO_4_, SO_2_). After pretreatment, the materials were enzymatically hydrolysed using a cellulolytic cocktail (Celluclast + Novozym188) supplemented with a hemicellulolytic cocktail (Ultraflo). Scanning electron microscopy and small angle X-ray scattering were utilized to evaluate the structural changes, of the differently steam pretreated materials, before and after the enzymatic hydrolysis.

**Results:**

Scanning electron microscopy revealed increased surface roughness and pore enlargement in all the materials after enzymatic hydrolysis. The higher the severity of the pretreatment, the more the surface was rough since it was easier for the enzymes to access the binding site. As revealed by small angle X-ray scattering (SAXS), increasing the enzymatic hydrolysis of hemicellulose did not result in further collapse of cellulose. In line with the SAXS result, a qualitative evaluation of the cellulose surface using Congo red showed a larger exposed cellulose surface area after enzymatic hydrolysis.

**Conclusions:**

The present study reports the microstructural changes caused by pretreatment and enzymatic hydrolysis of Norway spruce. By enzymatically increasing the hemicellulose hydrolysis, the exposed cellulose surface area increases meaning that the cellulose might be easier to access for the enzymes. Structural analysis of biomass after enzymatic hydrolysis can direct the choice of enzymes for improved saccharification efficiency.

**Supplementary Information:**

The online version contains supplementary material available at 10.1186/s13068-025-02616-7.

## Background

Lignocellulosic materials represent a potential cornerstone for a future bioeconomy. Their use in bioethanol production is already underway and they can serve as a source of other value-added products [1, 2]. However, their large-scale industrial utilization remains limited by some challenges such as the low efficiency of enzymatic hydrolysis [3, 4]. Numerous variables, including structure and composition of the plant cell wall, can influence the saccharification process [5–7]. Conifers cells are organized radially into ray cells and longitudinally into tracheids [8]. These two cell types are connected by pits: those that connect adjacent tracheids are known as bordered pits; whereas those that link tracheids with ray cells are known as cross-field pits [8]. Owing to such structural complexity, a pretreatment step is necessary to open up the conifers cell wall and increase the accessibility of enzymes to the corresponding binding sites [9, 10].

Among the several lignocellulosic biomasses, spruce is a challenging biomass to enzymatically hydrolyse mainly due to the lignin and hemicellulose composition [9–12]. There are different pretreatment technologies that can be used and each of them has positives and negatives aspects [9, 10]. In this work steam explosion was chosen as pretreatment since it is considered a mature and flexible technology. This treatment alters the material’s overall structure, as it induces breaks in the cell wall and reduces the size of wood chips, thereby facilitating enzyme accessibility to polysaccharides [10, 11]. Structural changes in the cell wall are due mainly to a sudden drop in pressure during pretreatment, which leads to rapid evaporation of superheated water. The sudden evaporation of adsorbed water triggers mechanical forces that push from the inside to the outside of wood chips, causing loss of size and structural disruption [11]. The cracks in the cell wall resulting from the steam pretreatment, change its shape and alter the connections between tracheids [13]. Damage is more evident on the edges of wood chips, but it can occur also internally if an acidic catalyst and high temperatures are used during pretreatment. Pielhop et al. [11] reported 10–100 nm holes on the surface of steam-pretreated spruce caused by the fast evaporation of the water during the pretreatment. During steam pretreatment, lignin depolymerizes/recondenses on the surface of cellulose fibres if temperatures surpass the glass transition point [14, 15].

Severe pretreatment conditions are required to obtain sufficiently high concentrations of fermentable sugars by subsequent enzymatic hydrolysis of spruce [10, 11]. The solubilization of hemicellulose and the production of secondary degradation compounds, which might prevent microbial fermentation, are the primary drawbacks of severe steam pretreatment. To limit the formation of degradation compounds, milder conditions can be applied; however, the resulting pretreated material has more compact structure and less accessible to enzymes. The reduced accessibility is related to the retention of hemicellulose in the solid fraction, but also to lower depolymerization/recondensation of lignin during pretreatment [16].

The efficiency of enzymatic hydrolysis is affected by structural features of the target biomass and enzymatic factors [17]. Structural changes of pretreated biomasses have been studied with respect to biomass composition, particle size, cellulose crystallinity and pore volume [17–19]. The action of enzymes on non-model or complex substrates such as lignocellulosic biomass has been poorly studied and the resulting structural changes remain largely unknown [20]. Understanding these enzymes factors are quite challenging due to the difficulty of studying the interactions between enzymes and complex substrates (such as lignocellulosic biomasses) [17].

The present study aimed to evaluate the structural changes occurring in mildly steam pretreated spruce after enzymatic hydrolysis. To obtain ultrastructural information, scanning electron microscopy (SEM) and small angle X-ray scattering (SAXS) were applied. Spruce was steam pretreated under six different conditions and enzymatically hydrolysed by cellulolytic (Celluclast® 1.5 L + Novozym® 188) and hemicellulolytic (Ultraflo®) cocktails. The spruce surface was imaged by SEM before and after steam pretreatment, as well as after enzymatic hydrolysis. Further changes to cellulose ultrastructure before and after enzymatic hydrolysis were detected by SAXS, while Congo red staining revealed the extent of the exposed cellulose surface.

## Materials and methods

### Lignocellulosic materials and enzymatic hydrolysis

Milled Norway spruce (*Picea abies*) chips were kindly provided by the Widtskövle sawmill. Steam pretreatment was performed as described elsewhere [16]. Briefly, the conditions were 180 °C without addition of an acid catalyst and 210 °C for 5 min, with/without addition of an acid catalyst, such as 1% (w/w) acetic acid (HAc), 0.1% (w/w) H_3_PO_4_, 0.1% (w/w) H_2_SO_4_ or 3% (w/w) SO_2_ (Table S1). These conditions were selected to vary the extent and composition of the remaining hemicellulose in the materials after pretreatment, as described elsewhere [16]. Carbohydrate composition of steam-pretreated materials (Table S2) was analysed following the National Renewable Energy Laboratory/TP-510–42,618 protocol and is reported elsewhere [21].

Enzymatic hydrolysis was carried out with three different cocktails: Celluclast® 1.5 L, Novozym® 188, and Ultraflo® (Novozymes, Lyngby, Denmark). Although the exact cocktails composition is not stated by the manufacturer, Celluclast® 1.5 L is defined as cellulolytic cocktail, Novozym® 188 contains mainly β-glucosidase activity and Ultraflo® contains endo-xylanases [22–24]. Reactions were performed in triplicates, in 2-mL screw-cap tubes, and for a total reaction weight of 1.8 g. The substrate was loaded at 2% w/w of dry mass (DM) and suspended in 0.15 M acetate buffer (pH 5). The reaction mixture was autoclaved at 121 °C for 20 min, after which the sterile-filtered enzyme mixtures were added aseptically at 10 filter paper units g^−1^ DM for Celluclast® 1.5 L and 10 U g^−1^ DM for Novozym® 188. Ultraflo® was loaded at twice the mannanase activity measured in Celluclast® 1.5 L. An adjusted version of the filter paper unit assay was used to quantify the activity of Celluclast® 1.5 L [25, 26]; whereas the β-glucosidase assay was used to quantify the activity of Novozym® 188 with some adjustments [16, 27]. The mannanase activity in Ultraflo® was measured as described elsewhere [28], with the release of reducing sugars from glucomannan quantified by the 3,5-dinitrosalicylic acid method [25]. The reactions were carried out at 40 °C for 48 h in an oven with a constant end-over-end rotation (Big S.H.O.T III™; Boekel Scientific, Feasterville, PA, USA) at 25 rpm. To terminate the reaction, the mixture was boiled for 10 min. The samples were then stored at −20 °C. Sugar release was measured by isocratic HPAEC-PAD (ICS-5000; Dionex, Sunnyvale, CA, USA) using a Carbopac PA1 column as described elsewhere [29]. The conversion of polymers to soluble monomeric sugars after enzymatic hydrolysis at low solids loading was calculated as explained by Zhu et al. [30].

### SEM ultrastructural analysis

SEM analyses were performed using a JEOL 7800F Prime (Jeol Inc., Tokyo, Japan) at high vacuum (10^–4^ Pa) and an acceleration voltage of 2 kV. Samples were mounted on an aluminium stub with silver paint to increase conductivity and coated with a 5-nm gold layer.

### Focused ion beam (FIB) measurements

Substrate cross-sections were analysed on a combined SEM–FIB instrument (FIB—FEI Versa3D LoVac; FEI, Hillsboro, OR, USA). The ion beam was used to remove the material, while the electron beam was used to image the cross-section. The ion beam was applied at 30 kV with 65 nA for the trench and 2 nA for polishing. The electron beam was applied at 2 kV. Samples were mounted on an aluminium stub with silver paint to increase conductivity. A 500-nm-thick gold layer was deposited on the specimen to protect the surface underneath from being damaged by the ion beam while also increasing conductivity. The coincidence point of the ion and electron beams was 52°, with 10-mm working distance.

### SAXS measurements

SAXS curves were recorded using a Mat:Nordic system (SAXSLAB, Copenhagen, Denmark) equipped with a Rigaku 003 + high-brilliance microfocus Cu-radiation source (Wilmington, MA, USA). The X-ray wavelength was 1.54 Å^−1^ and the measured q-range was 0.01–0.68 Å^−1^. A silver behenate sample was quantified before every new set of measurements to calibrate the q-axis. Wet specimens were placed in sandwich cells covered with mica windows, sealed, and measured at room temperature. Measurements were performed on three different specimens before and after enzymatic hydrolysis. The two-dimensional scattering patterns were radially averaged using SAXSGui software. One-dimensional SAXS curves were then plotted as so-called Kratky plots (q vs Iq2). The maxima of the peak (q max) observed in Kratky plots at around 0.1 Å^−1^ corresponded to the fibril-to-fibril distance [31, 32]. Peak maxima were determined by fitting the Gaussian functions. The dimensions (d) were then calculated using Eq. 1:

1 $$d = \frac{2\pi }{{q\max }}.$$

### Cellulose surface area evaluation

Duplicates of the different steam-pretreated substrates were enzymatically hydrolysed with and without Ultraflo® supplementation to Celluclast® 1.5 L + Novozym® 188 as previously explained. After enzymatic hydrolysis, the mixtures were boiled at 100 °C for 10 min to inactivate the enzymes. The hydrolysed materials were washed with 1% w/w Tween80 to remove any residual enzymes. For this, the samples were incubated at 45 °C for 2 h (Big S.H.O.T III™), after which the supernatant was removed by centrifugation at 13,000 × *g* for 2 min. The hydrolysed materials were stained with Congo red as described previously [33–35] with some adjustments. Briefly, 6 mg mL^−1^ of Congo red (Sigma Aldrich, St. Louis, MO, USA) was diluted 1:2 in 0.06 M phosphate buffer (pH 6) containing 2.8 mM NaCl and added to _~_2% w/w of dry hydrolysed material. The reactions were carried out at 60 °C for 24 h in an oven with constant end-over-end rotation (Big S.H.O.T III™). After incubation, the mixture was centrifuged at 7000 × *g* for 2 min, the absorbance of the supernatant from samples and the reference solution was measured at 498 nm, and the amount of adsorbed dye was calculated based on the fraction of glucan in the biomass after pretreatment (Table S2).

## Results and discussion

In the present work, Norway spruce was steam pretreated under six different conditions [16], consisting of two different temperatures (180 °C and 210 °C) plus various catalysts (HAc, H_3_PO_4_, H_2_SO_4_, and SO_2_) (Table S1). The substrates subjected to each of the above treatments were denoted as follows: *STEX*_180°C/auto_, *STEX*_210°C/auto_, *STEX*_210°C/HAc_, *STEX*_210°C/H3PO4_, *STEX*_210°C/H2*SO*4_, and *STEX*_210°C/*SO*2_. The structure and composition of the materials after pretreatment are differently affected by the various pretreatment conditions, as previously investigated [16]. Briefly, surface defibrillation and lignin depolymerization were observed increasing pretreatment severity. Cellulose analysis revealed the rearrangement of microfibrils leading to the collapse of cellulose microfibrils and therefore to the formation of larger microfibril aggregates during the pretreatment. This microfibril rearrangement likely contributed to the observed increase in enzymatic hydrolysis yields as better enzyme accessibility resulted.

### Surface and cross-section evaluation of untreated and steam-pretreated spruce

**Fig. 1:**
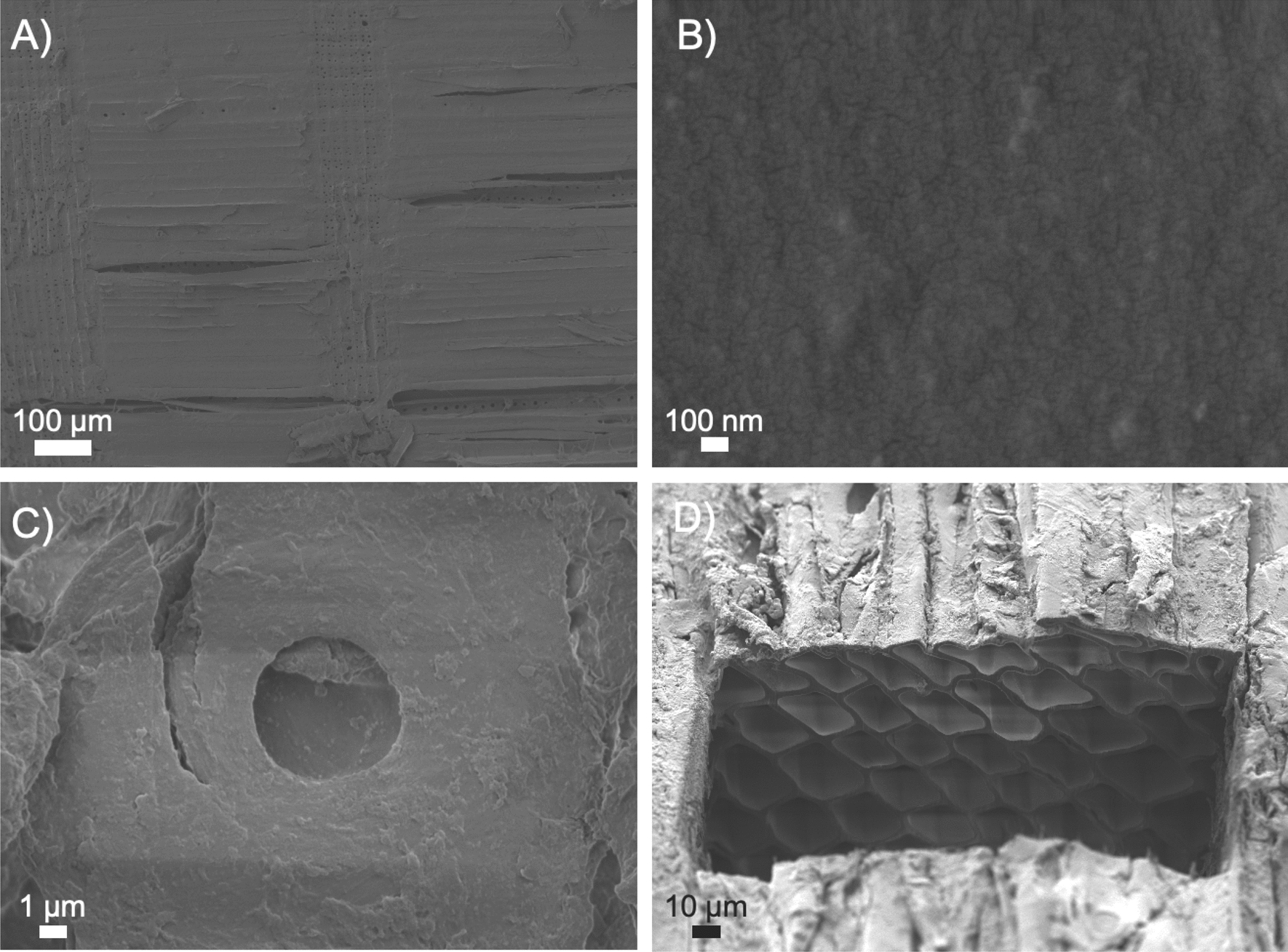
SEM images obtained using the secondary electron signal from untreated Norway spruce wood at **A** low magnification (100 ×) and **B** high magnification (50,000 ×). **C** Detail of a bordered pit obtained by SEM. **D** Internal tracheid structure revealed in a cross-section obtained by combined SEM–FIB

SEM was used to visually compare the surface and porosity of steam-pretreated spruce with respect to untreated material (Figure 1). At low magnification (100×), a well-defined structure, with visible tracheids (“longitudinal lines”) and ray cells (“perpendicular lines”) was clearly visible (Figure 1A). At a higher magnification (50000×), the surface appeared flat and compact (Figure 1B). The smaller cracks observed on the surface were due to a 5-nm gold coat sputtered during sample preparation. Figure 1C shows an intact and well-rounded bordered pit, whose shape is due to the curving of cellulose microfibrils [36]. Combined SEM–FIB imaging of cross-section cuts revealed the internal organization of tracheids (Figure 1D).

**Fig. 2:**
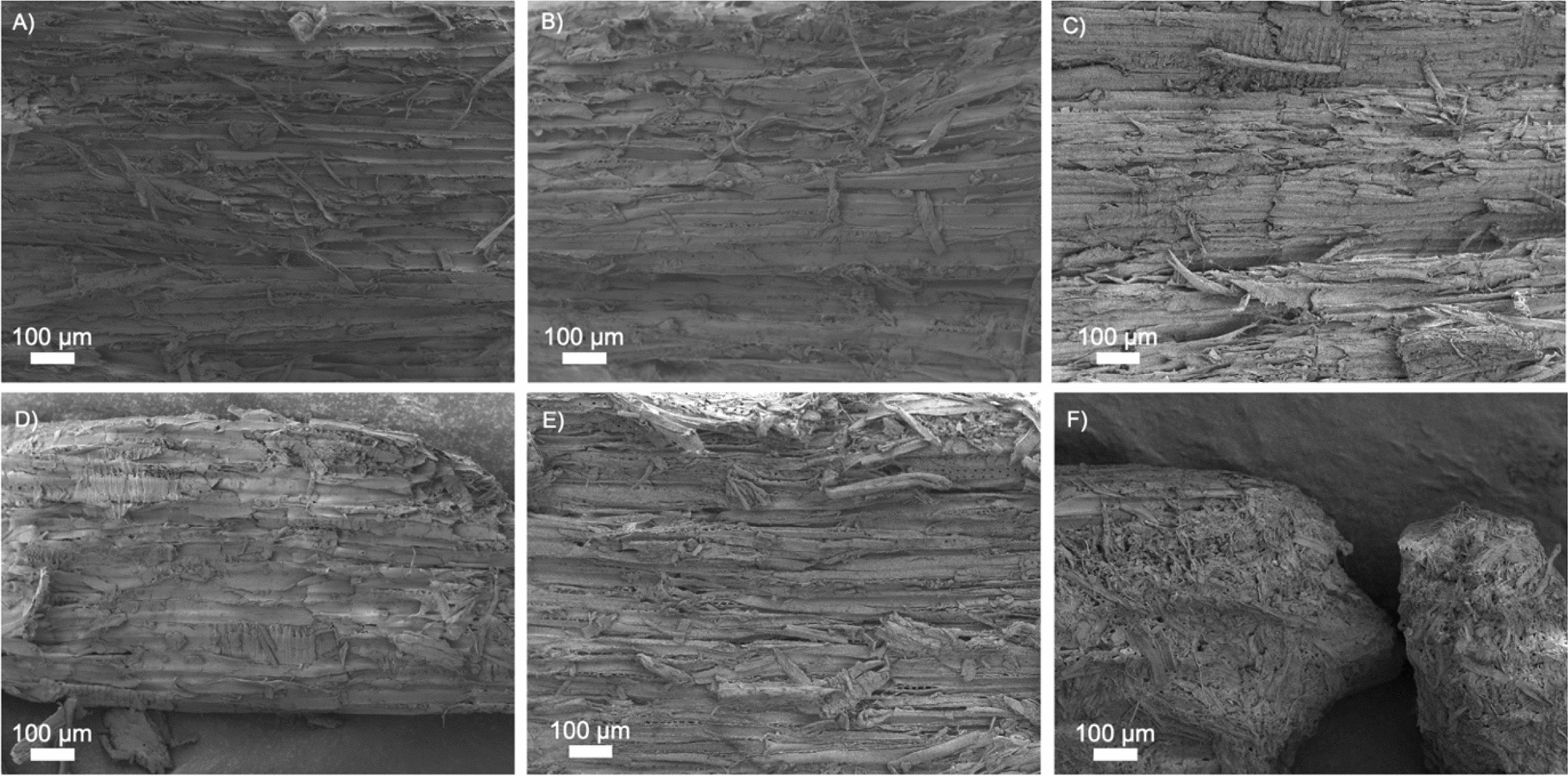
SEM images obtained at low magnification (100 ×) using the secondary electron signal from Norway spruce wood subjected to different pretreatments. **A** STEX_180°C/auto_, **B** STEX_210°C/auto_, **C** STEX_210°C/HAc_, **D** STEX_210°C/H3PO4_, **E** STEX_210°C/H2SO4_, and **F** STEX_210°C/SO2_

Low-magnification (100 ×) SEM images of steam-pretreated materials are presented in Fig. 2. Clear cracks between tracheids can be observed in all substrates, and an increase in surface defibrillation can be noted with increasing severity of pretreatment. No clear difference can be detected between samples *STEX*_180°C/auto_ (Fig. 2A) and *STEX*_210°C/auto_ (Fig. 2B), due to the small difference in severity of the pretreatment (Table S1). In the absence of a catalyst, steam pretreatment relies on the natural release of acetyl groups from galactoglucomannan on hemicellulose (only 1 acetyl group every 3–4 hexose units) [12, 37]. Addition of a catalyst during pretreatment alters profoundly the structure of lignocellulosic biomass. *STEX*_210°C/HAc_, *STEX*_210°C/H3*PO*4_, and *STEX*_210°C/H2SO4_ samples displayed a more defibrillated surface, characterized by numerous large cracks (Fig. 2C–E). Such changes are linked to greater hemicellulose solubilization, lignin depolymerization/recondensation, and cellulose microfibrils rearrangement [16]. In the *STEX*_210°C/SO2_ sample, which was subjected to a highly severe pretreatment (Table S1), hemicellulose was completely solubilized (Table S2) while the effect on the cellulose and lignin was greater compared to the other catalysed materials [16]. For these reasons the material after the pretreatment has an almost disrupted structure (Fig. 2F).

**Fig. 3:**
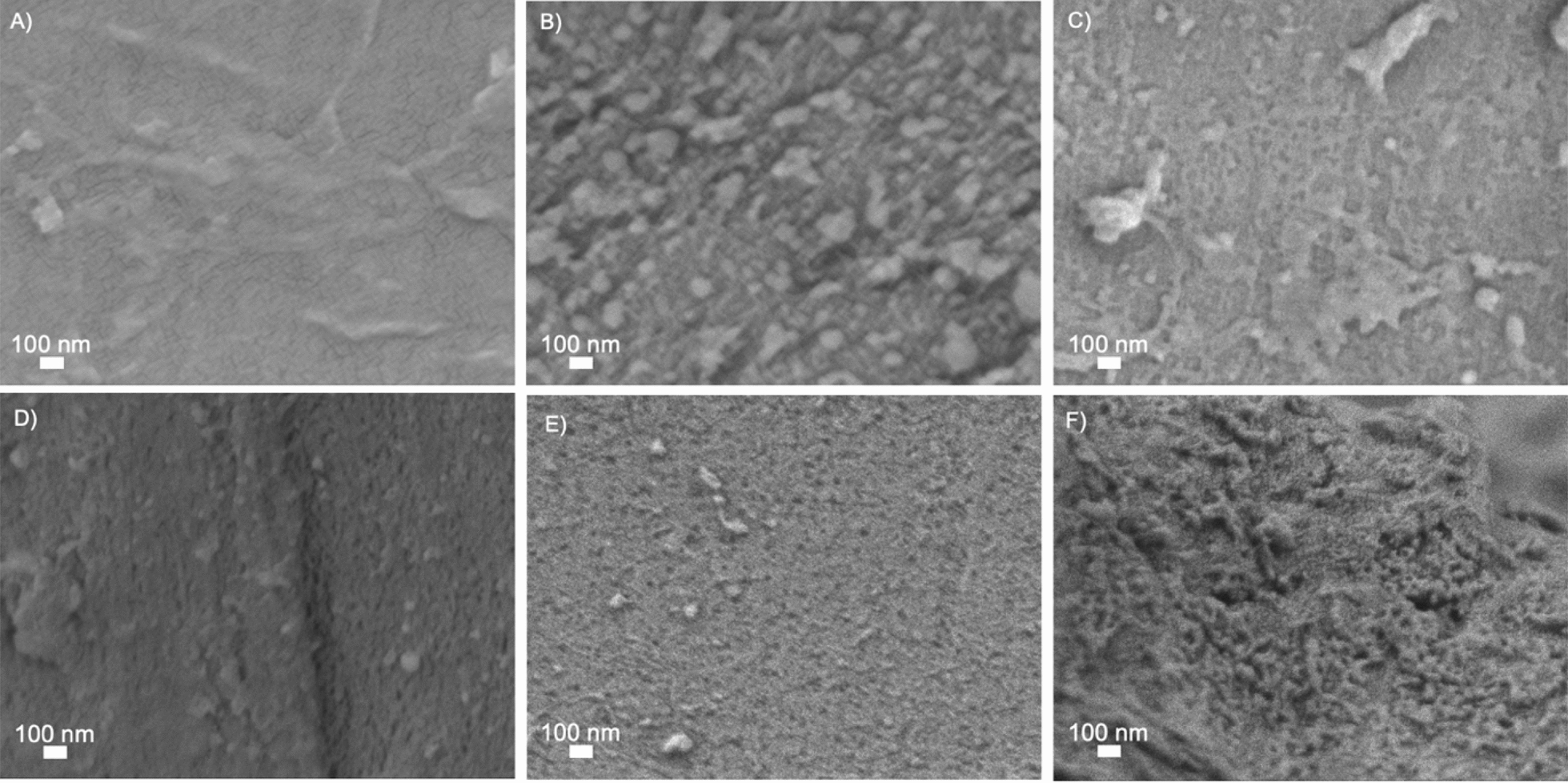
SEM images obtained at high magnification (50,000 ×) using the secondary electron signal from Norway spruce wood subjected to different pretreatments. **A** STEX_180°C/auto_, **B** STEX_210°C/auto_, **C** STEX_210°C/HAc_, **D** STEX_210°C/H3PO4_, **E** STEX_210°C/H2SO4_, and **F** STEX_210°C/SO2_

High-magnification (50,000 ×) images of all pretreated materials are presented in Fig. 3. Surface roughness augmented dramatically with increasing severity of pretreatment. Spruce samples subjected to the least severe conditions (*STEX*_180°C/auto_) exhibited a compact and flat surface (Fig. 3A). The temperature used during pretreatment (180 °C) is close to the lignin transition glass temperature (140–160 °C) [38], suggesting that lignin likely failed to recondense and form droplets on the surface [39]. Under more severe conditions, lignin tends to depolymerize and recondense completely [40].

In contrast, samples *STEX*_210°C/auto_, *STEX*_210°C/HAc_, *STEX*_210°C/H3PO4_, and *STEX*_210°C/H2SO4_ (Fig. 3B–E) displayed increased surface roughness with increasing severity of pretreatment. A more pronounced roughness, compared to the *STEX*_180°C/auto_, could be linked to relocation of lignin to the surface, as well as to hemicellulose solubilization. At 210 °C, there is a larger depolymerization/recondensation of lignin on the surface [38]. Also, when an acid catalyst is added during pretreatment, it favours the solubilization of hemicellulose, which no longer acts as a spacer between cellulose microfibrils and leads to their rearrangement [16]. Sample *STEX*_210°C/SO2_ (Fig. 3F) exhibited an extremely rough surface compared to all other materials. Boarded pits (Figure S1) were also imaged after pretreatment. In line with previous literature [8], the presence of cracks was observed in the pits of materials subjected to pretreatment with a catalyst.

**Fig. 4:**
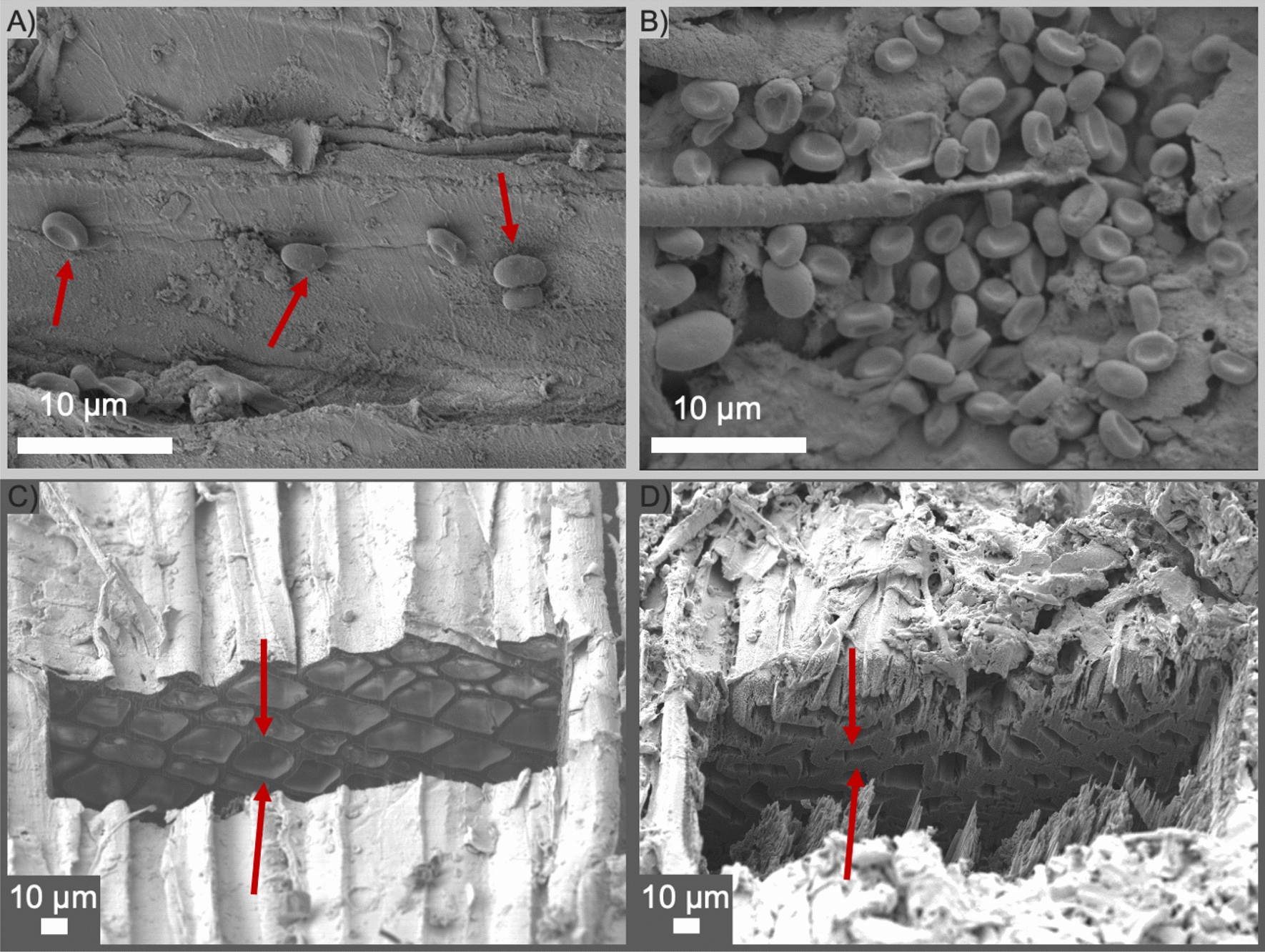
SEM images obtained using the secondary electron signal from Norway spruce wood subjected to different pretreatments. Lignin droplets are clearly visible (red arrows) on the surface of **A** *STEX*_*210°C/auto*_ and **B** *STEX*_*210°C/SO2*_ samples. Low-magnification SEM images obtained using the secondary electron signal of FIB cross-sections reveal the internal structure of tracheids in **C** *STEX*_*210°C/auto*_ and **D** *STEX*_*210°C/SO2*_ samples. The collapsed cell walls of tracheids are highlighted with red arrows

SEM images of the surface were obtained to gain a deeper knowledge of the presence of the lignin droplets on the surface (Fig. 4A, B) while SEM–FIB imaging of cross-section cuts revealed the internal organization of tracheids (Fig. 4C, D). Lignin droplets were detected on the surface of both *STEX*_*210°C/auto*_ (Fig. 4A) and *STEX*_*210°C/SO2*_ (Fig. 4B) samples, although they were more numerous and larger in the latter. These two samples were chosen because they represent two extremes of pretreatment conditions that were used in the present work. As mentioned before, depolymerization and recondensation of lignin on the surface is linked to both the transition glass temperature and to the catalyst used during pretreatment. As severity of the latter increases, the frequency of lignin droplets augments [14, 15]. A comparison between the cross-sections of *STEX*_*210°C/auto*_ (Fig. 4C) and *STEX*_*210°C/SO2*_ (Fig. 4D) samples revealed a more collapsed structure in the latter (highlighted by red arrows); whereas the internal structure of the former was very similar to untreated material (Fig. 1D). Overall, SEM and FIB images show a strong agreement between the internal and superficial features of steam-pretreated samples (Figs. 2, 3, 4). This can be explained by complete solubilization of hemicellulose and larger effect on the lignin in the *STEX*_*210°C/SO2*_ sample, which leads to the collapse of tracheid cell walls.

### Enzymatic hydrolysis of steam-pretreated spruce

**Fig. 5:**
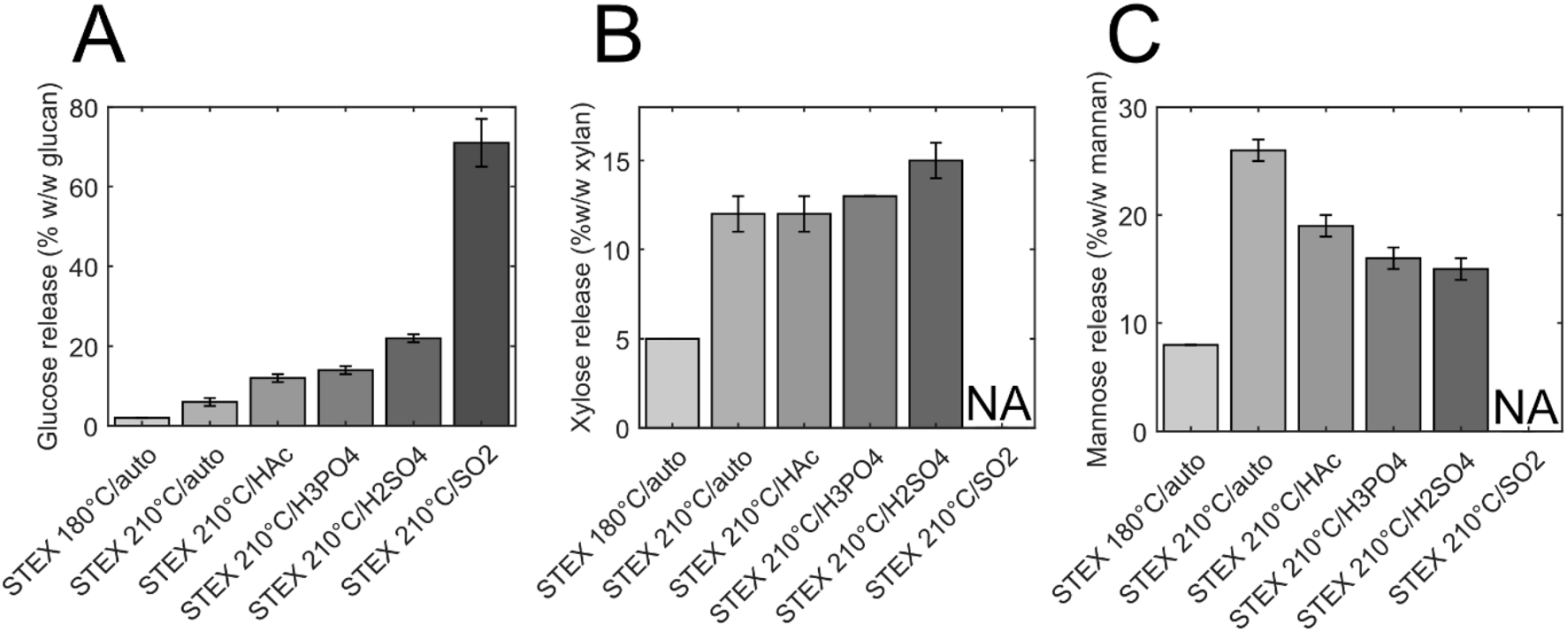
Enzymatic hydrolysis of steam-pretreated spruce. Release of **A** glucose, **B** xylose, and **C** mannose after 48 h of hydrolysis with Celluclast® 1.5 L, Novozym® 188, and Ultraflo®. The grey scale used in the bar plots denotes the corresponding increase in the severity of pretreatment. Data represent the mean ± standard deviation of triplicate measurements

To investigate the action of enzymes on the structure of various steam-pretreated spruce materials, enzyme-catalysed sugar release was assessed. Previously, enzymatic hydrolysis of steam-pretreated softwood using Celluclast® 1.5 L and Novozym® 188 resulted in low amount of sugars being released from hemicellulose [16]. Therefore, in the present work, these two commercial cocktails were supplemented with Ultraflo® to improve hemicellulose hydrolysis and evaluate the ensuing structural changes. Glucose was released from both cellulose and galactoglucomannan (Fig. 5). The release of glucose increased from 2% w/w DM in the *STEX*_180°C/auto_ sample to 22% w/w DM in *STEX*_210°C/H2SO4_ and 71% w/w DM in *STEX*_210°C/SO2_ (Fig. 5A). Xylose, which was released from the arabinoglucuronoxylan backbone on hemicellulose, reached 15% w/w DM in the *STEX*_210°C/H2SO4_ sample (Fig. 5B); whereas mannose released from the galactoglucomannan backbone (26% w/w DM) was maximal in the *STEX*_210°C/auto_ sample (Fig. 5C). Residual hemicellulose after catalyst-mediated pretreatment is harder to hydrolyse compared to that in autocatalysed materials [16]. Here, supplementation with Ultraflo® favoured the release of xylose and particularly mannose.

### Surface and cross-section evaluation of steam-pretreated spruce after enzymatic hydrolysis

**Fig. 6:**
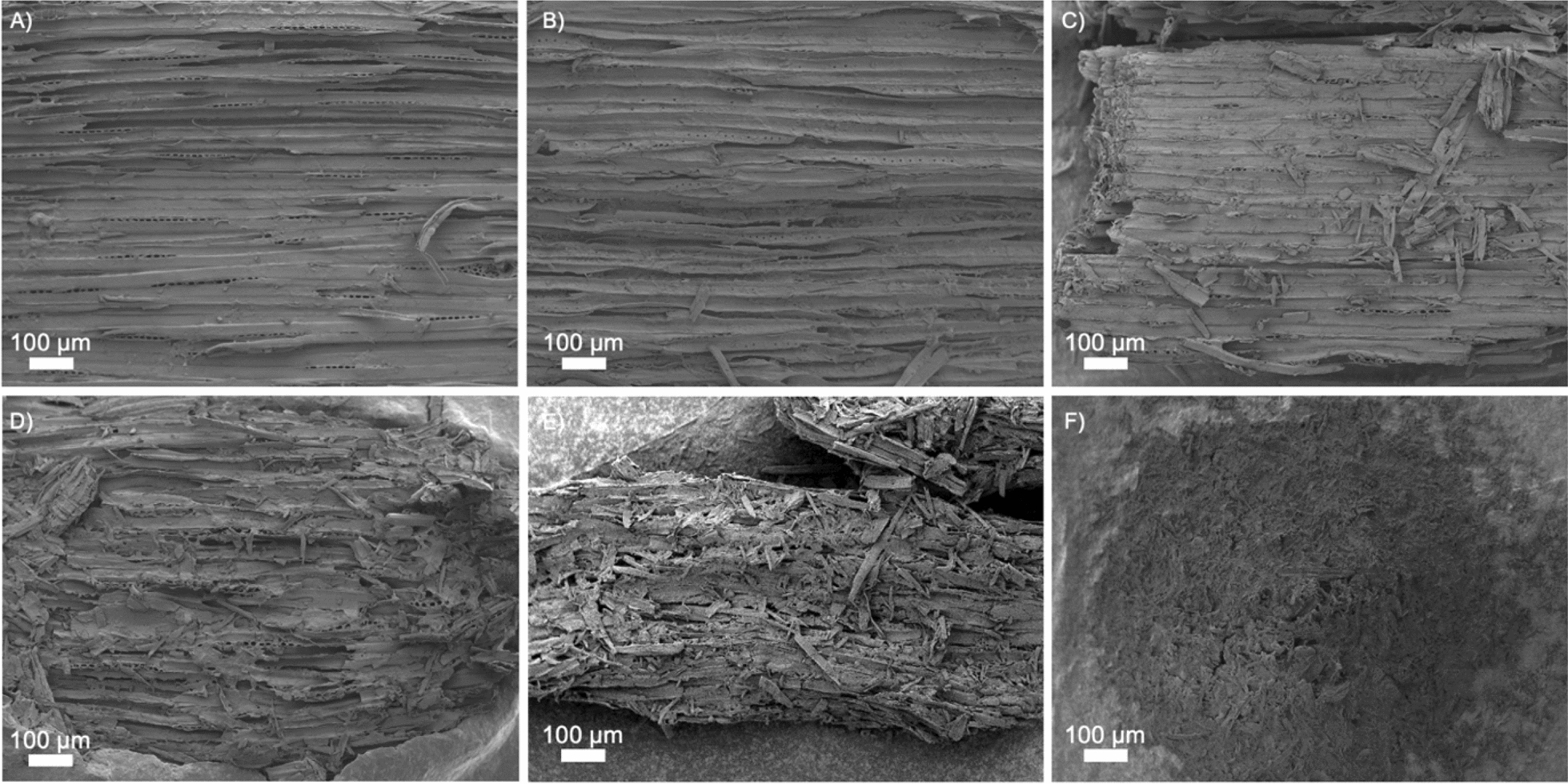
SEM images obtained at low magnification (100 ×) using the secondary electron signal from Norway spruce wood subjected to different pretreatments and subsequent enzymatic hydrolysis. **A** *STEX*_*180°C/auto*_, **B** *STEX*_*210°C/auto*_, **C** *STEX*_*210°C/HAc*_, **D** *STEX*_*210°C/H3PO4*_, **E** *STEX*_*210°C/H2SO4*_, and **F** *STEX*_*210°C/SO2*_

The impact of enzymatic hydrolysis on the surface of steam-pretreated spruce was assessed by SEM and is summarized in Fig. 6 (100 × magnification). A qualitative comparison between low-magnification (100 ×) SEM images reporting changes caused by enzymatic hydrolysis (Fig. 6) and surface changes after pretreatment (Fig. 2) shows that enzymatic hydrolysis had different effects depending on pretreatment severity. In the absence of catalyst (*STEX*_180°C/auto_ and *STEX*_210°C/auto_) all fibres present on the surface after pretreatment were successfully degraded by the enzymes (Fig. 6A, B). In the presence of a catalyst (*STEX*_210°C/HAc_, *STEX*_210°C/H3PO4_, and *STEX*_210°C/H2SO4_) enzymatic degradation was more pronounced showing a more disrupted surface than the two autocatalysed materials (Fig. 6C–E). Severe pretreatment conditions facilitated enzyme access to the binding sites and caused greater structural disruption. Indeed, in the *STEX*_210°C/SO2_ sample, the structure was not visible anymore as it was entirely degraded (Fig. 6F). Therefore, the observed structural changes suggest greater enzymatic hydrolytic action with increasing severity of pretreatment, as also reported in literature [11, 41–43].

**Fig. 7:**
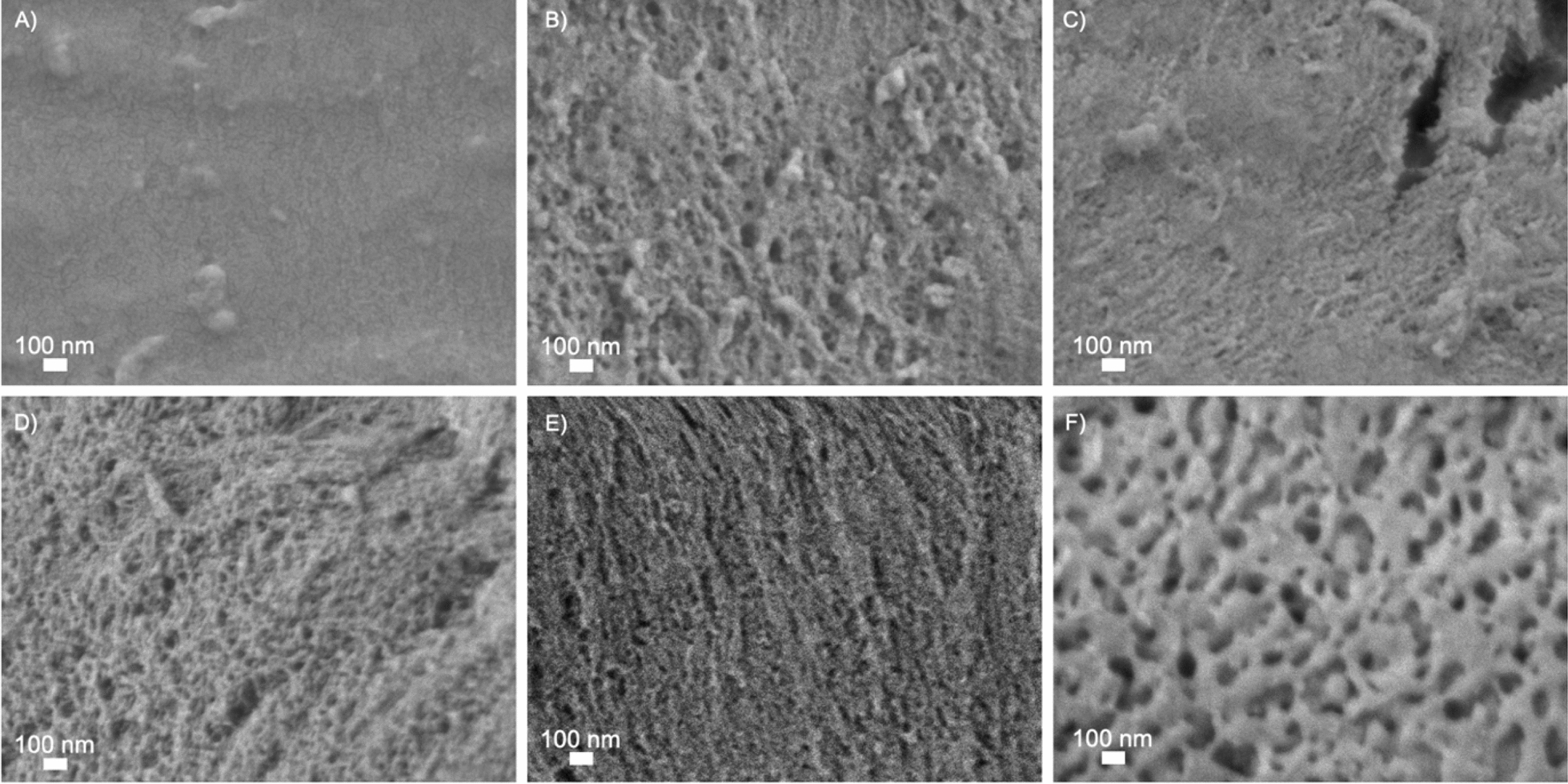
SEM images obtained at high magnification (50,000 ×) using the secondary electron signal from Norway spruce wood subjected to different pretreatments and subsequent enzymatic hydrolysis. **A** *STEX*_*180°C/auto*_, **B** *STEX*_*210°C/auto*_, **C** *STEX*_*210°C/HAc*_, **D** *STEX*_*210°C/H3PO4*_, **E** *STEX*_*210°C/H2SO4*_, and **F** *STEX*_*210°C/SO2*_

A qualitative comparison between high-magnification (50,000 ×) SEM images reporting changes caused by enzymatic hydrolysis (Fig. 7) and surface changes elicited by pretreatment (Fig. 3) revealed that the enzymes’ primary impact was to increase roughness and create/enlarge pores. Again, the effect of the enzymes varied depending on the severity of pretreatment. For lowest severely pretreated samples (*STEX*_180°C/auto_), the surface looked very similar after saccharification (Fig. 7A) as following pretreatment only (Fig. 3A). Accordingly, the enzymes accessed and degraded only the fibres sticking out from the surface. This hypothesis is in line with the low yield of sugars released from the *STEX*_180°C/auto_ sample after enzymatic hydrolysis (Fig. 5). Hemicellulose and lignin are not significantly affected by pretreatments conditions used for the *STEX*_*180°C/auto*_ sample and, as a result, the enzymes’ accessibility to the material is diminished [16]. The *STEX*_*210°C/SO2*_ sample represents the other extreme, whereby hemicellulose is fully solubilized following pretreatment. Compared to the surface of pretreated sample (Fig. 3F), enzymatic hydrolysis of the *STEX*_210°C/SO2_ substrate resulted in a very rough surface with enlarged pores (Fig. 7F). The *STEX*_210°*C/SO*2_ is more accessible to enzymes and easier to degrade, as confirmed also by high glucose release (Fig. 5). Therefore, increased pretreatment severity induces substantial structural changes that improve enzyme accessibility and, consequently, sugar release.

A comparison of the materials *STEX*_210°C/auto_, *STEX*_210°C/Hac_, *STEX*_210°C/H3PO4_, and *STEX*_210°C/SO2_ before (Figure 3B–E) and after enzymatic hydrolysis (Figure 7B–E) revealed that the enzymatic hydrolysis enhanced surface roughness and pore size. Even though the surface of *STEX*_210°C/auto_, *STEX*_210°C/Hac_, *STEX*_210°C/H3PO4_, and *STEX*_210°*C/H*2*SO*4_ samples did not differ significantly after enzymatic hydrolysis, there was nevertheless an appreciable difference in sugar release (Figure 5). Such discrepancy can be explained by the importance of structural parameters on enzymatic hydrolysis [17]. The cellulose ultrastructure, lignin’s chemical state, pore size, surface area, and biomass composition may all affect enzyme accessibility and, consequently, hydrolysis yields [17, 39]. Indeed, lowering the crystallinity of cellulose increases the accessibility of enzymes and boosts hydrolysis yields [39]. Hemicellulose removal has also been shown to increase cellulose accessibility, as it no longer acts as a barrier together with the lignin [44, 45]. Overall, hemicellulose composition and lignin condensation exert a strong effect on cellulose ultrastructure [16].

### Cellulose ultrastructural changes due to further hemicellulose hydrolysis

**Fig. 8:**
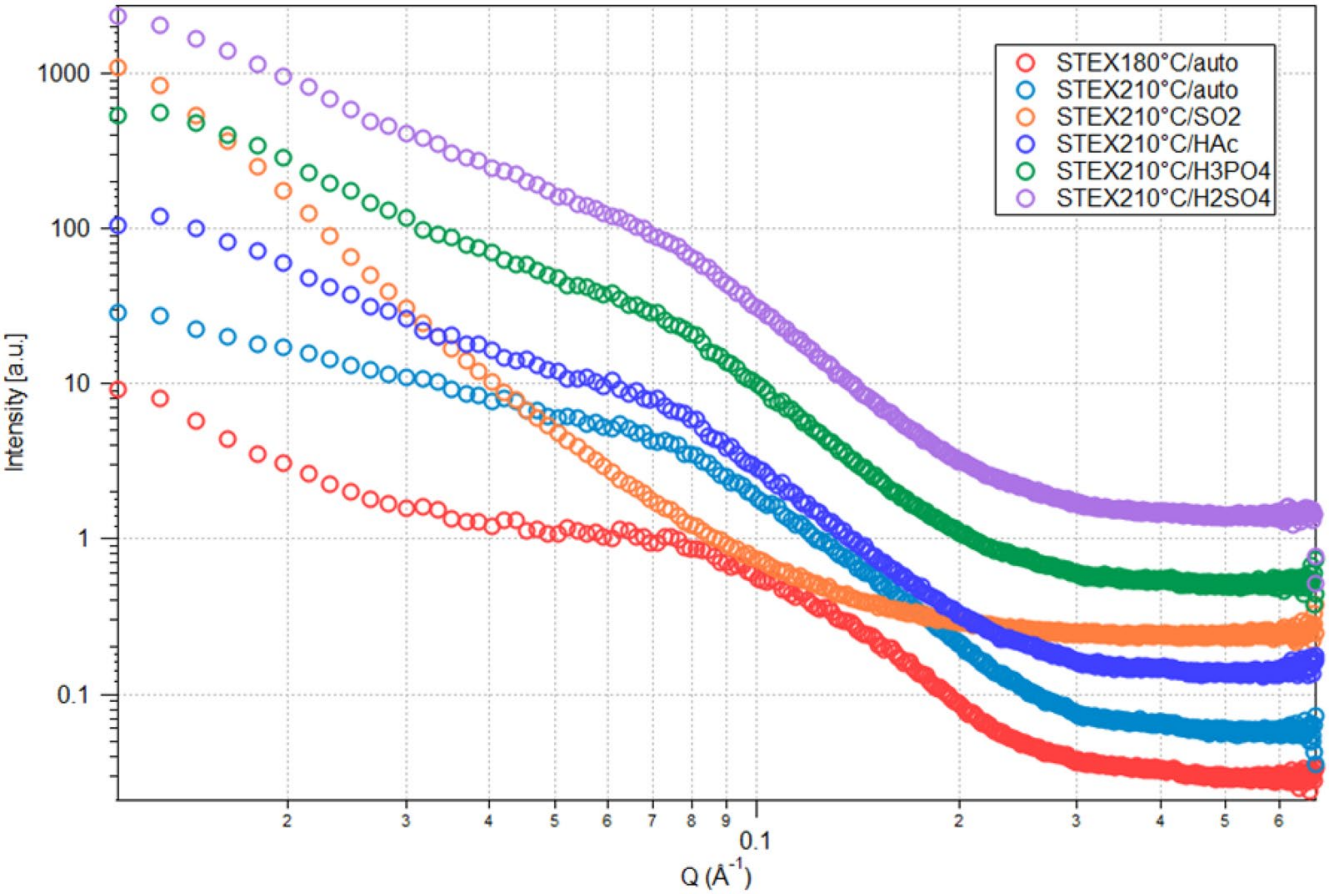
SAXS curves of pretreated spruce samples subjected to enzymatic hydrolysis: *STEX*_*180°C/auto*_, *STEX*_*210°C/auto*_, *STEX*_*210°C/HAc*_, *STEX*_*210°C/H3PO4*_, *STEX*_*210°C/H2SO4*_, and *STEX*_*210°C/SO2*_. Scattering curves were shifted for visual clarity

**Fig. 9:**
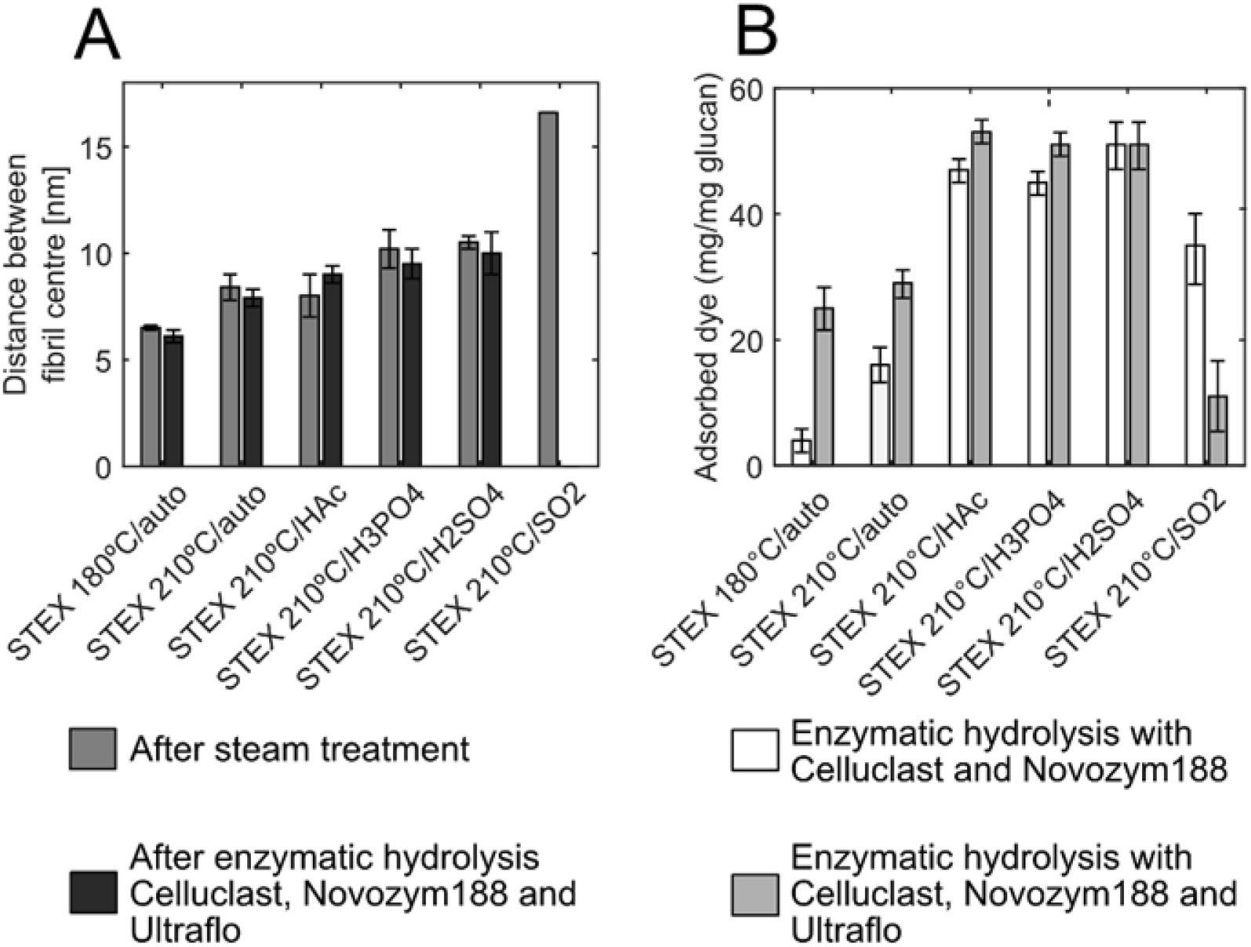
Cellulose ultrastructure evaluation before and after enzymatic hydrolysis with or without Ultraflo® supplementation. **A** The distance between cellulose fibril centres in the different steam-pretreated materials before (light grey bars) and after (dark grey bars) enzymatic hydrolysis with Celluclast® 1.5 L, Novozym® 188, and Ultraflo® was assessed using SAXS. Values are presented as average with standard deviation of three measurements, and were obtained from the Gaussian fits extracted from Kratky plots. **B** Adsorbed Congo red in the different steam-pretreated materials after enzymatic hydrolysis with (dark grey bars) or without (light grey bars) Ultraflo®. Values are presented as average of duplicates and the average of the absolute deviations from the mean

SEM images of differently pretreated spruce samples before and after enzymatic hydrolysis (Figs. 2, 3, 6, and 7) showed increased enzyme accessibility to the substrate at increasing severity of the pretreatment. To assess the effect of greater enzymatic hemicellulose hydrolysis on enzyme accessibility, the cellulose ultrastructure was investigated by SAXS. SAXS data provide valuable insights on the arrangement of microfibrils within plant cells. In a typical wood sample, a distinct peak is often observed at approximately 0.11 Å^−1^ (Fig. 8). The dimensions of this peak correlate with the distance between microfibrils [31, 32]. Notably, the distance determined by SAXS might refer also to the distance between cellulose microfibril aggregates, rather than individual microfibrils. This interpretation is supported by the tight packing of microfibrils within these aggregates, which leads to similar scattering patterns. Enzymatic hydrolysis augmented the distance between fibril centres (Fig. 9A), as indicated by a shift in the peak to lower q-values. Unlike for other substrates, the scattering curve of the *STEX*_*210°C/SO2*_ sample lacked the characteristic peak corresponding to disrupted or nonhomogeneous structures arising from enzymatic hydrolysis. Changes to cellulose ultrastructure after pretreatment have been attributed to hemicellulose solubilization during steam explosion [16, 45]. In contrast, further degradation of hemicellulose during enzymatic hydrolysis did not lead to further collapse of the cellulose. This could mean that by enzymatically increasing the hemicellulose hydrolysis there is an increase of the exposed cellulose surface area.

The cellulose surface area is another determinant that affects the enzymatic saccharification in lignocellulosic materials, because increased cellulose surface area results in greater enzyme accessibility [19, 46]. Here, the available cellulose surface area was qualitatively evaluated using Congo red dye since it specifically binds the exposed cellulose surface. After incubation with the different materials, the absorbance of the dye in the supernatant was spectrophotometrically measured that can be related to the amounts of dye bound to the materials. The more dye is bound to the material the more cellulose is exposed [33–35]. Specifically, this method was used to determine if an increase in hemicellulose hydrolysis contributed to greater cellulose exposure. Figure 9B reports the amount of Congo red adsorbed by the different steam-pretreated materials after enzymatic hydrolysis in the presence or absence of Ultraflo®. Supplementation with a hemicellulolytic enzyme had a greater effect in the two autocatalysed materials (*STEX*_180°*C/*auto_ and *STEX*_210°*C/*auto_), as indicated by an appreciable increase in the amount of dye bound when Ultraflo® was added. With increased severity of pretreatment, the effect of hemicellulase supplementation became less evident. The diminished impact of addition of hemicellulytic enzymes on acid-catalysed materials can be attributed to the paucity of residual hemicellulose after such treatment [16]. Thus, the enzymatically increase in hemicellulose hydrolysis does not help expose more cellulose. In the material subjected to the harshest pretreatment (STEX_210°*C/*SO2_), the overall amount of dye bound to the material was lower than in any other circumstance, including when Ultraflo® was added. According to the literature, Congo red may not offer an accurate measure of cellulose surface area in harshly pretreated biomass [34]. Wiman et al. [34] implied Congo red to quantify the total cellulosic surface of the steam-pretreated spruce while Simons’ staining was employed to analyse the accessible surface for the enzymes. The result was that increasing severity of pretreatment the resulted material did not have in larger total cellulosic surface areas (Congo red) but rather in a larger accessible cellulosic surface area (Simons’ stain) due to larger pores on the cellulose surface.

## Conclusions

A deeper understanding of how the structure of spruce changes during pretreatment and enzymatic hydrolysis would benefit the scaling up of bioethanol production from softwood. In this work, six distinct steam pretreatment conditions were applied to spruce wood, including variations in temperature (180 °C and 210 °C) and catalysts (HAc, H_3_PO_4_, H_2_SO_4_, and SO_2_). These pretreatment conditions significantly altered the wood’s structure and, subsequently, its susceptibility to enzymatic saccharification. High-magnification SEM images, through qualitative analysis, revealed a clear correlation between pretreatment severity and increased surface roughness. The roughening of the surface was likely a result of lignin redistribution to the surface and the solubilization of hemicellulose, which normally serves as a spacer between cellulose microfibrils. In the most severe pretreatment condition (*STEX*_*210°C/SO2*_), hemicellulose was completely solubilized, leading to a significant collapse of the overall structure.

The next phase of the study explored how enzymatic hydrolysis, facilitated by the addition of Ultraflo® to Celluclast® 1.5 L and Novozym® 188, further impacted the structure of the biomass. SEM images, through qualitative analysis, showed not only an increase in surface roughness, but also an enlargement of the pores after enzymatic treatment. The severity of pretreatment directly influenced the degree of surface roughness, thereby enhancing enzyme accessibility. Importantly, SAXS analysis revealed that while enzymatic hydrolysis improved cellulose accessibility, it did not cause additional collapse of the cellulose microfibrils, suggesting a more efficient exposure of cellulose fibres for hydrolysis.

The findings presented in this study contribute to a deeper understanding of biomass saccharification. The increase in surface roughness and cellulose accessibility due to pretreatment and enzymatic hydrolysis is crucial for optimizing enzymatic efficiency and achieving higher sugar yields. Furthermore, the results provide critical insights into how pretreatment conditions influence enzymatic activity, pointing to potential strategies for improving saccharification processes. These findings pave the way for future research aimed at refining pretreatment strategies and enzymatic formulations.

## Supplementary Information

Additional file1.

## Data Availability

No datasets were generated or analysed during the current study.
