# Supplementary material for: Structural changes and cellulose ultrastructure mapped with electron microscopy and SAXS after enzymatic hydrolysis of mildly steam pretreated Norway spruce
Source: Biotechnol Biofuels Bioprod. 2025 Feb 21;18:19. doi: 10.1186/s13068-025-02616-7 (PMC11846163; doi:10.1186/s13068-025-02616-7)
Supplement: Supplementary file 1 — Additional file1. [file 13068_2025_2616_MOESM1_ESM.docx]

Structural changes and cellulose ultrastructure mapped with electron microscopy and SAXS after enzymatic hydrolysis of mildly steam pretreated Norway spruce

Maria E. F. Brollo^1‡^, Fabio Caputo^2‡^, Polina Naidjonoka^3^, Lisbeth Olsson^2^*^,^*^4^, Eva Olsson^1*^

^1^Department of Physics, Chalmers University of Technology, Gothenburg – Sweden

^2^Division of Industrial Biotechnology, Department of Life Sciences, Chalmers University of

Technology, Gothenburg - Sweden.

^3^  Division of Materials Physics, Department of Physics, Chalmers University of Technology, Kemigården 1, 412 96, Gothenburg, Sweden

^4^Wallenberg Wood Science Center, Chalmers University of Technology,

Gothenburg - Sweden

*E-mail: eva.olsson@chalmers.se

**Present address: Yangi AB, Varberg, Sweden<mailto:fabioca@chalmers.se>

‡ Maria E.F. Brollo and Fabio Caputo share first authorship

| **Table S1.** Summary of the conditions applied for pretreatment of Norway spruce biomass and combined severity factor [1]. | | | | | | | |
| --- | --- | --- | --- | --- | --- | --- | --- |
|  | **STEX_180°C/auto_** | | **STEX_210°C/auto_** | **STEX_210°C/HAc_** | **STEX_210°C/H3PO4_** | **STEX_210°C/H2SO4_** | **STEX_210°C/SO2_** |
| Temperature  (°C) | 180 | 210 | | 210 | 210 | 210 | 210 |
| Residence time (min) | 5 | 5 | | 5 | 5 | 5 | 5 |
| Catalyst^a^ | autocatalyzed | autocatalyzed | | 1% HAc | 0.1% H_3_PO_4_ | 0.1% H_2_SO_4_ | 3% SO_2_ |
| pH liquid fraction | 3.9 | 3.6 | | 3.1 | 2.9 | 2.5 | 1.5 |
| CSF | 0.7 | 1.0 | | 1.5 | 1.7 | 2.1 | 3.1 |
| ^a^The percentages are expressed in w/w. | | | | | | | |
| HAc, acetic acid. | | | | | | | |

| **Table S2.** Compositional analysis of lignocellulosic compounds in spruce before and after pretreatment. Data represent mean values ± standard deviation of triplicates. The glucan amount corresponds to the amount of glucose from cellulose and from galactoglucomannan [1]. | | | | |
| --- | --- | --- | --- | --- |
|  | **Raw material (%w/w DM)** | **STEX_180°C/auto_ (%w/w DM)** | **STEX_210°C/auto_ (%w/w DM)** | **STEX_210°C/SO2_ (%w/w DM)** |
| Glucan | 45.6 ± 0.7 | 49 ± 1 | 52 ± 2 | 50 ± 2 |
| Xylan | 5.0 ± 0.1 | 4.9 ± 0.6 | 2.4 ± 0.1 | 0.0 ± 0.0 |
| Arabinan | 0.5 ± 0.0 | 0.0 ± 0.0 | 0.0 ± 0.0 | 0.0 ± 0.0 |
| Galactan | 1.7 ± 0.1 | 1.5 ± 0.1 | 0.2 ± 0.0 | 0.0 ± 0.0 |
| Mannan | 12.1 ± 0.1 | 10.7 ± 0.6 | 4.1 ± 0.2 | 0.0 ± 0.0 |
| ASL | 2.6 ± 0.1 | 2.3 ± 0.1 | 1.4 ± 0.0 | 1.3 ± 0.0 |
| AIL | 32.4 ± 0.1 | 34.4 ± 0.6 | 41.4 ± 0.4 | 51.4 ± 0.5 |
| Ash | BDL | BDL | BDL | BDL |
| Recovery | 100 ± 1 | 103 ± 2 | 101 ± 1 | 103 ± 2 |
|  | | | | |
|  |  | **STEX_210°C/HAc_ (%w/w DM)** | **STEX_210°C/H3PO4_ (%w/w DM)** | **STEX_210°C/H2SO4_ (%w/w DM)** |
| Glucan |  | 60.5 ± 0.3 | 61.3 ± 0.4 | 61.0 ± 0.1 |
| Xylan |  | 1.7 ± 0.0 | 1.3 ± 0.0 | 0.7 ± 0.0 |
| Arabinan |  | 0.0 ± 0.0 | 0.0 ± 0.0 | 0.0 ± 0.0 |
| Galactan |  | 0.1 ± 0.0 | 0.1 ± 0.0 | 0.1 ± 0.0 |
| Mannan |  | 1.2 ± 0.0 | 0.9 ± 0.0 | 0.6 ± 0.0 |
| ASL |  | 2.9 ± 0.1 | 2.8 ± 0.1 | 2.7 ± 0.1 |
| AIL |  | 36.0 ± 0.1 | 36.9 ± 0.1 | 38.2 ± 0.4 |
| Ash |  | BDL | BDL | BDL |
| Recovery |  | 103 ± 1 | 103 ± 1 | 103 ± 1 |
| AIL, acid-insoluble lignin; ASL, acid-soluble lignin; BDL, below detection limit. | | | | |

**Analysis of pit structure**

Analysis of the pits and the areas around them revealed damage and cracks, whose intensity increased with the severity of pretreatment. When steam is released during the explosion phase, the pits represent an easy escape route for the gas; however, their thin walls become broken in the process. Similar fractures, along with damaged or collapsed pit membranes, were observed by Zhang and Cai [2] in subalpine fir when applying steam explosion.


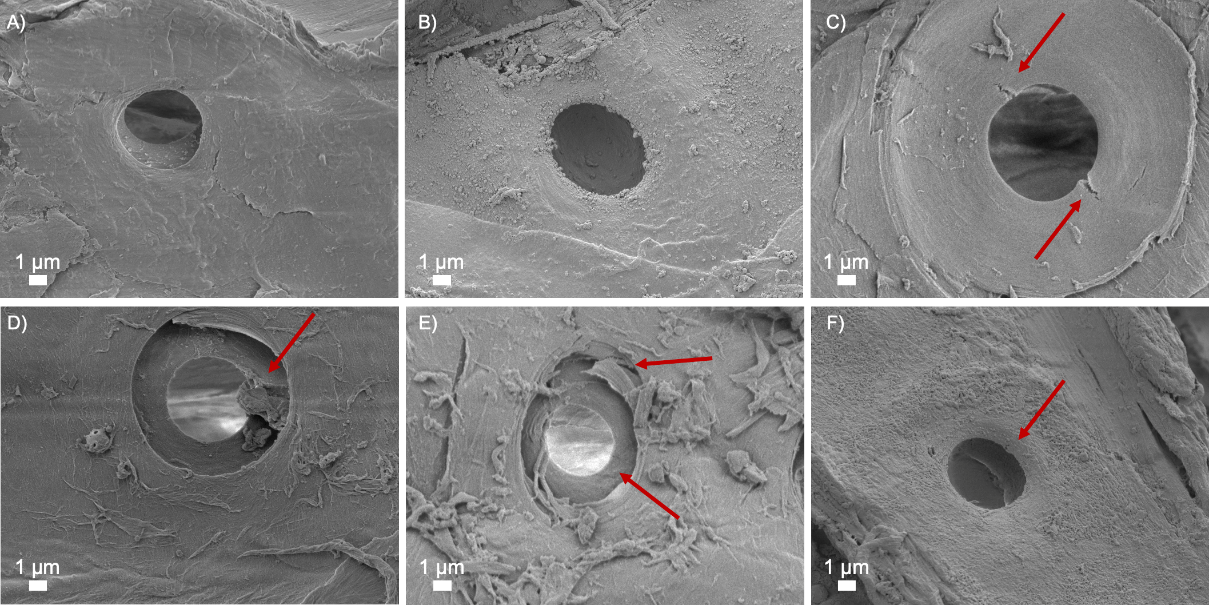


Figure S1: Pit structure as seen by low-magnification SEM images obtained using the secondary electron signal from Norway spruce wood subjected to different pretreatments. Small fractures are highlighted by red arrows. (A) STEX_180°C/auto_, (B) STEX_210°C/auto_, (C) STEX_210°C/HAc_, (D) STEX_210°C/H3PO4_, (E) STEX_210°C/H2SO4_, and (F) STEX_210°C/SO2_.

References

1. Caputo, F., Al-Rudainy, B., Naidjonoka, P., Wallberg, O., Olsson, L., Novy, V. : Understanding the impact of steam pretreatment severity on cellulose ultrastructure, recalcitrance, and hydrolyzability of Norway spruce. Biomass Conversion and Biorefinery, (2022).
2. Zhang, Y., Cai, L.: Effects of steam explosion on wood appearance and structure of sub-alpine fir. Wood Science and Technology (2006).
